# Descriptive Analysis of Real-World Data on Fingolimod Long-Term Treatment of Young Adult RRMS Patients

**DOI:** 10.3389/fneur.2021.637107

**Published:** 2021-03-03

**Authors:** Tjalf Ziemssen, Holger Albrecht, Judith Haas, Luisa Klotz, Michael Lang, Christoph Lassek, Stephan Schmidt, Benjamin Ettle, Ulf Schulze-Topphoff

**Affiliations:** ^1^Zentrum für klinische Neurowissenschaften, Universitaetsklinikum Carl Gustav Carus Dresden, Dresden, Germany; ^2^Praxis für Neurologie, Munich, Germany; ^3^Zentrum für Multiple Sklerose, Juedisches Krankenhaus Berlin, Berlin, Germany; ^4^Klinik für Allgemeine Neurologie, Universitätsklinikum Muenster, Muenster, Germany; ^5^Nervenärztliche Gemeinschaftspraxis, Ulm, Germany; ^6^Neurologische Gemeinschaftspraxis Kassel und Vellmar, Kassel, Germany; ^7^Neurologische Gemeinschaftspraxis Schmidt, Neudecker, Viebahn & Kronenberger, Bonn, Germany; ^8^Novartis Pharma GmbH, Nuremberg, Germany

**Keywords:** RRMS, fingolimod, young adults, real-world evidence, early treatment, long-term study

## Abstract

**Background:** Fingolimod (Gilenya®) is approved for adult and pediatric patients with highly active relapsing–remitting multiple sclerosis (RRMS).

**Objectives:** The objective was to describe the effectiveness of fingolimod in young adults compared to older patients in clinical practice.

**Methods:** PANGAEA is the largest prospective, multi-center, non-interventional, long-term study evaluating fingolimod in RRMS. We descriptively analyzed demographics, MS characteristics, and severity in two subgroups of young adults (≤20 and >20 to ≤30 years) and older patients (>30 years).

**Results:** Young adults had lower Expanded Disability Status Scale (EDSS) scores compared to older patients (1.8 and 2.3 vs. 3.2) at baseline. The mean EDSS scores remained stable over 5 years in all subgroups. Young adults had higher annual relapse rates (2.0 and 1.7 vs. 1.4) at study entry, which were reduced by approximately 80% in all subgroups over 5 years. The proportion of patients with no clinical disease activity in year 4 was 52.6 and 73.4 vs. 66.9% in patients ≤20, >20 to ≤30 years and >30 years, respectively. The symbol digit modalities test score increased by 15.25 ± 8.3 and 8.3 ± 11.3 (mean ± SD) from baseline in patients >20 to ≤30 and >30 years.

**Conclusions:** Real-world evidence suggests a long-term treatment benefit of fingolimod in young RRMS patients.

## Introduction

Relapsing multiple sclerosis (MS) represents a continuous spectrum of disease ranging from clinically isolated syndrome over relapsing–remitting multiple sclerosis (RRMS) to secondary progressive MS (SPMS) (1). Most RRMS patients are diagnosed at an age of 30–40 years (2), but some patients show early onset of MS at a childhood age or as young adults (3). The disease characteristics in these patients differ from adult MS patients, e.g., in pediatric MS, the relapse rate was shown to be two to three times higher, and pediatric MS patients often experience more severe relapses (4, 5). Despite increased relapse severity, pediatric patients often recover completely (5, 6). With respect to disability, cognitive dysfunction is typically more frequent in pediatric compared to adult patients, while locomotor disability is less pronounced (3, 7). Therefore, the time to disability milestones as measured by the Expanded Disability Status Scale (EDSS) might be longer in younger patients. Due to the early onset of the disease, these milestones are still reached at a younger age (3). Overall, these features suggest that MS in younger patients is even more characterized by inflammatory processes than in older patients. Despite differences in disease characteristics and limited efficacy data in younger MS patients, in general, the same treatment regimens should be used for adults, young adults, and even children. However, the treatment armamentarium for the latter group is limited, as not all MS drugs are approved for use in children.

Fingolimod (Gilenya®, Novartis Pharma AG) was first approved in 2011 as a once-daily oral treatment for adult patients with RRMS and since then has gained marketing authorization in over 80 countries. Approximately 296,700 patients have been treated with fingolimod in both the clinical trial and post-marketing settings, and the total patient exposure now exceeds 746,700 patient-years. In 2019, it has also gained approval for the treatment of children and adolescents with RRMS. Its efficacy and safety in pediatric patients had been investigated in the PARADIGMS study, in which fingolimod was shown to be more effective than treatment with interferon-beta 1a (8). The subgroup analyses of three pivotal studies in adults (FREEDOMS, FREEDOMS II, and TRANSFORMS) have shown benefits of fingolimod treatment over placebo and beta-interferons in terms of relapse prevention and MRI activity in young adults (9). However, data on the use of fingolimod in young adults is limited to a small number of patients in the respective study populations.

In the present analysis of the PANGAEA study (Post-Authorization Non-interventional German SAfety of GilEnyA in RRMS patients), the effectiveness of fingolimod in young adults in real-world settings was investigated. PANGAEA was a non-interventional study recruiting RRMS patients from 2011 to 2013 to assess long-term safety, tolerability, effectiveness, and patient-reported outcomes of fingolimod under real-life conditions (10–12) for an observational period of (maximum) 5 years.

Here we report the results of a descriptive analysis of fingolimod treatment in PANGAEA subgroups of young adult patients with RRMS (≤20 and >20–30 years of age) treated up to 5 years in daily clinical practice. A subgroup of the PANGAEA population with age above 30 years is used as the reference cohort.

## Patients and Methods

### Study Design

PANGAEA was a prospective, multi-center, non-interventional, long-term study of fingolimod (0.5 mg) for the treatment of patients with RRMS (10). It was conducted in Germany, including office-based neurologists and neurology clinics. Patients who received fingolimod according to the summary of product characteristics were eligible. The treatment followed a common clinical routine, and the observation period was *a priori* set to up to 60 months. Follow-up visits were documented about every 3 months. Recruitment into the study started in April 2011 and finished in December 2013, with a total of 4,229 patients, of whom 4,032 were included in the analyses. Data included baseline characteristics (sex, age, body mass index) and MS characteristics (disease duration, number of relapses in the past year). Disease severity using EDSS, severity symbol digit modalities test (SDMT), multiple sclerosis severity score (MSSS), and annual relapse rate (ARR) was analyzed every year for the observational period of 60 months. Due to the non-interventional study design, assessments followed clinical practice routine and were optional. The present subgroup analysis of PANGAEA data comprises young adults, i.e., patients ≤20 years as well as patients >20 to ≤30 years of age in comparison to patients >30 years of age.

### Administrative Procedures

The study was conducted according to the current recommendations for observational studies of the following institutions: the Voluntary Self-Control of Pharmaceutical Companies Codex (FSA-Codex), the Federal Institute for Drugs and Medical Devices and the Paul-Ehrlich-Institut (PEI), and the Research-Based Pharmaceutical Companies (vfa). Prior to study initiation, an ethics committee was consulted, and the study was notified to the competent higher federal authority, the Federal Association of Statutory Health Insurance Physicians and the Statutory Health Insurance. Patients were only included after providing written informed consent at the time of the baseline visit.

### Statistical Methods

The presented data are part of analyses conducted in January 2020. All data were analyzed descriptively using SAS, version V9.4. Analysis of baseline characteristics included demographics, disease history, and prior treatment. The endpoints of interest were treatment interruptions, annual relapse rate, EDSS changes, SDMT changes, clinical disease activity as defined by relapses and disability development, as well as effectiveness and tolerability as reported by physicians and patients. In addition to a baseline score-referenced analysis of EDSS progression, a roving EDSS analysis approach was used (13). As this was a non-interventional study, no visit windows were defined and no rules for handling of missing or incomplete data were established. Therefore, instead of exact EDSS assessment dates, the follow-up visit schedule has been used for roving EDSS analysis. Assessments at month 1 follow-up visit were not included. Furthermore, missing EDSS values were not imputed and had no impact on the analysis.

A methodological limitation of this descriptive analysis is that correction for confounding factors like disease duration was not possible because of the strong correlation between this factor and age. Due to the relatively low number of patients in the youngest age group, propensity score matching would have resulted in a comparison of individual cases instead of a representative group comparison. The present results therefore should be interpreted with caution, especially with respect to disease characteristics that depend on the disease duration.

All analyses were performed by age subgroups with the following cutoffs: <20 years, >20 to ≤30 years, and >30 years. Continuous data were analyzed as mean and standard deviation, while categorical data were analyzed as absolute and relative frequencies.

## Results

The present analysis included 81 patients younger than 20 years of age (2.0% of the total population), 819 patients aged 20–30 (20.3%) years, and 3,130 patients older than 30 years of age (77.6%). The gender distribution was similar between age groups. Young adults included in PANGAEA had a shorter disease duration on average (2.8 and 4.5 vs. 9.3 years) and lower EDSS and MSSS scores compared to patients older than 30 years (EDSS: 1.8 and 2.3 vs. 3.2; MSSS: 4.7 and 5.0 vs. 5.2). Although the EDSS scores were higher in older age groups, the SDMT scores were similar in patients aged 20–30 years and patients older than 30 years (SDMT: 45.5 vs. 45.6; SDMT was not assessed in patients <20 years). The annual relapse rate within 12 and 24 months prior to study inclusion was higher in younger patients (2.0 and 1.7 vs. 1.4 and 2.8 and 2.6 vs. 2.1), and the proportion of relapse-free patients within 12 months before study inclusion was smaller (6.3 and 15.1 vs. 21.5%). Time from first symptoms to diagnosis was 0.6 and 0.7 years in young adults, compared to 2.2 years in patients older than 30 years. The proportion of patients with concomitant diseases increases by age (16.0 and 24.1 vs. 34.8%). The most frequent diseases in all age groups were psychiatric disorders (3.7, 5.5, and 9.9%) and nervous system disorders (2.5, 6.3, and 8.9%), with a higher total frequency in older patients (Table 1).

**Table 1:** Patients' characteristics, disease history, and pretreatment at baseline.

**Mean ± SD, unless otherwise specified**	**≤20 years** ***N* = 81**	**>20 to ≤30 years** ***N* = 900**	**>30 years** ***N* = 3,130**
Female, *n* (%)	*N*′= 81 63 (77.8)	*N*′= 819 590 (72.0)	*N*′= 3,130 2,247 (71.8)
Age (years)	*N*′= 81 19.1 ± 1.0	*N*′= 819 26.2 ± 2.7	*N*′= 3,130 42.9 ± 7.6
Height (cm)	*N*′= 55 167.5 ± 9.5	*N*′= 608 171.7 ± 8.3	*N*′= 2,451 171.4 ± 8.8
Weight (kg)	*N*′= 54 65.2 ± 12.9	*N*′= 615 72.2 ± 17.7	*N*′= 2,446 75.0 ± 17.1
BMI	*N*′= 54 23.3 ± 4.1	*N*′= 602 24.3 ± 5.2	*N*′= 2,417 25.5 ± 5.2
Time since diagnosis (years)	*N*′= 77 2.8 ± 2.1	*N*′= 795 4.5 ± 3.1	*N*′= 2,911 9.3 ± 6.5
Time from first symptoms to diagnosis (years)	*N*′= 71 0.6 ± 1.9	*N*′= 686 0.7 ± 1.6	*N*′= 2,362 2.2 ± 4.1
Number of MS relapses within the last 12 months	*N*′= 79 2.0 ± 1.1	*N*′= 799 1.7 ± 1.3	*N*′= 3,079 1.4 ± 1.1
Number of MS relapses within the last 24 months	*N*′= 79 2.8 ± 1.4	*N*′= 800 2.6 ± 1.9	*N*′= 3,069 2.1 ± 1.6
Patients without relapse within the last 12 months, %	*N*′= 79 6.3	*N*′= 819 15.1	*N*′= 3,079 21.5
Patients with ≤1 relapse within the last 24 months, %	*N*′= 79 15.2	*N*′= 819 28.9	*N*′= 3,069 38.1
Total EDSS	*N*′= 74 1.8 ± 1.3	*N*′= 762 2.3 ± 1.5	*N*′= 2,875 3.2 ± 1.7
Total MSSS	*N*′= 71 4.7 ± 2.7	*N*′= 741 5.0 ± 2.6	*N*′= 2,691 5.2 ± 2.6
Total SDMT	Not assessed	*N*′= 26 45.5 ± 13.6	*N*′= 189 45.6 ± 13.6
Prior treatment, %	*N*′= 81	*N*′= 819	*N*′= 3,130
None	0.6	6.1	6.2
Beta interferons	63.0	53.1	47.4
Glatiramer acetate	18.5	22.8	23.6
Natalizumab	11.1	15.9	19.0
Mitoxantrone	0.0	0.7	1.5
Azathioprine	0.0	0.1	1.0
Missing	1.2	1.2	1.3
Most frequent (≥2% in any age group) concomitant diseases by SOC, %	*N*′= 81	*N*′= 819	*N*′= 3,130
Any concomitant disease	16.0	24.1	34.8
Psychiatric disorders	3.7	5.5	9.9
Investigations	2.5	1.2	2.3
Metabolism and nutrition disorders	2.5	2.6	4.6
Nervous system disorders	2.5	6.3	8.9
Vascular disorders	0.0	2.2	8.9
Endocrine disorders	0.0	1.8	4.1
Musculoskeletal and connective tissue disorders	0.0	1.6	3.5
General disorders and administration site conditions	1.2	1.5	2.9
Renal and urinary disorders	0.0	0.5	2.2
Respiratory, thoracic, and mediastinal disorders	1.2	1.3	2.0

*BMI, body mass index; EDSS, Expanded Disability Status Scale; MSSS, Multiple Sclerosis Severity Score; n, number of patients in the category; N, number of patients in the total analysis population; N′, number of patients with available data; SD, standard deviation; SOC, system organ class*.

About half of the patients in all three age groups completed the 5-year observational period on therapy (48.2 and 43.0 vs. 53.8%). Study discontinuations were more frequently related to a lack of effectiveness in the youngest group (9.5 and 6.2 vs. 5.6%), while disease progression or relapse (4.8 and 10.1 vs. 9.8%), patient wish (7.1 and 30.4 vs. 31.3%), and adverse events (7.1 and 13.3 vs. 26.0%) were more frequently reported as reason for discontinuation in the older subgroups of patients (numbers given for patients ≤20 years and >20 to ≤30 years vs. >30 years, respectively; multiple responses per patient included).

The mean annual relapse rate was reduced by ~70% in the first year and over 80% in the fifth year in all patient subgroups. The proportion of relapse-free patients increased by ~15% in all three age groups from year 1 to year 5 (Table 2).

**Table 2:** Annual relapse rate and proportion of relapse-free patients.

	**≤20 years** ***N* = 81**	**>20 to ≤30 years** ***N* = 819**	**>30 years** ***N* = 3,130**
**ANNUAL RELAPSE RATE, ARR** **±** **SD (N')**			
Baseline	2.0 ± 1.13 (79)	1.70 ± 1.33 (688)	1.40 ± 1.09 (3,067)
Year 1	0.65 ± 0.86 (68)	0.48 ± 0.80 (688)	0.41 ± 0.71 (2,658)
Year 2	0.63 ± 0.91 (52)	0.35 ± 0.64 (538)	0.31 ± 0.62 (2,207)
Year 3	0.48 ± 0.87 (42)	0.31 ± 0.65 (416)	0.25 ± 0.55 (1,834)
Year 4	0.47 ± 0.76 (32)	0.27 ± 0.65 (341)	0.20 ± 0.49 (1,575)
Year 5	0.38 ± 0.59 (21)	0.24 ± 0.55 (248)	0.20 ± 0.46 (1,134)
**RELAPSE-FREE PATIENTS, % (N')**			
Baseline (year−1)	6.3 (79)	15.1 (819)	21.5 (3,079)
Year 1	52.9 (68)	66.0 (688)	69.7 (2,658)
Year 2	57.7 (52)	72.5 (538)	75.4 (2,207)
Year 3	69.1 (42)	77.2 (416)	80.0 (1,834)
Year 4	65.6 (32)	80.9 (341)	83.1 (1,575)
Year 5	66.7 (21)	81.1 (248)	82.5 (1,134)

*ARR, annual relapse rate; N, number of patients in the total analysis population; N′, number of patients with available data*.

**Figure 1:**
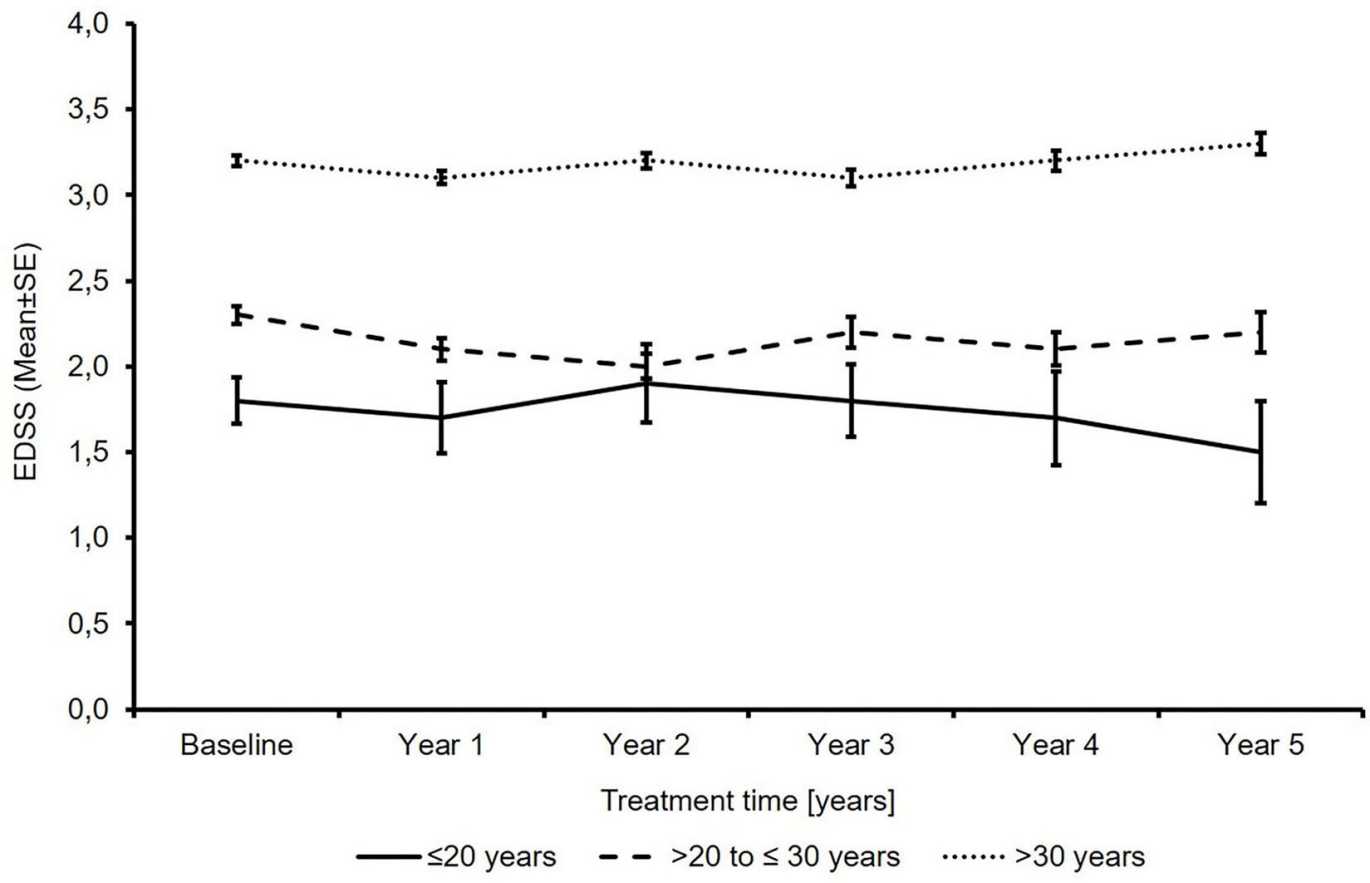
Total expanded disability status scale score (*N*′ ≤20 years: 74/53/37/27/18/13; *N*′ >20 to ≤30 years: 762/538/409/297/243/184; *N*′ >30 years: 2875/2076/1655/1295/1082/834; baseline/year 1/year 2/year 3/year 4/year 5).

**Figure 2:**
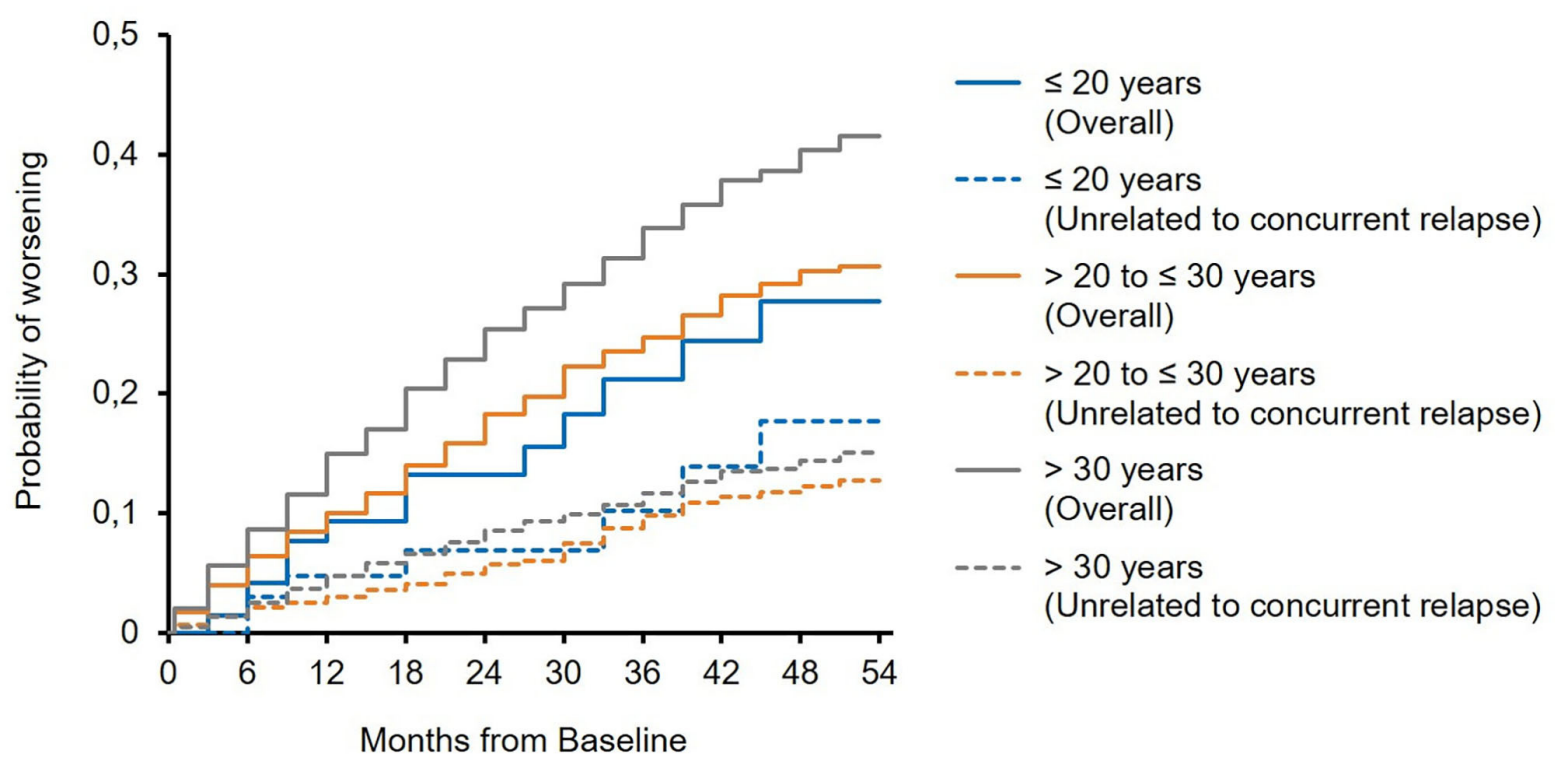
Roving expanded disability status scale (EDSS) progression overall and unrelated to concurrent relapse (EDSS worsening is related to a relapse if at least one relapse occurred between 30 days before start of EDSS worsening and 30 days after confirmation of EDSS worsening).

The mean EDSS score remained almost stable in all subgroups over 5 years of treatment (Figure 1). In 23.4 and 19.1 vs. 17.2% of the patients, sustained EDSS improvement was documented in year 4 of the observation period, while 11.8 and 10.6 vs. 14.7% had 6 months of confirmed disability progression as measured by EDSS. In 64.7 and 70.2 vs. 68.1% of the patients, the EDSS score remained stable. In 4 years of treatment, the proportion of patients without clinical disease activity (defined as the absence of EDSS progression and relapse) increased to 52.6 to 73.4 vs. 66.9% (for patients ≤20 years and >20 to ≤30 years *vs*. >30 years, respectively; Table 3). The analysis using a roving EDSS approach shows a higher cumulative probability of EDSS worsening after 4 years in patients >30 years compared to younger patients (Figure 2). All age groups had a similar cumulative probability of EDSS progression unrelated to relapse activity after 4 years (Figure 2).

**Table 3:** EDSS change and clinical disease activity per year (not aggregated).

	**≤20 years** ***N*** **=** **81**				**>20 to** **≤30 years** ***N*** **=** **819**				**>30 years** ***N*** **=** **3,130**			
**Year**	**1**	**2**	**3**	**4**	**1**	**2**	**3**	**4**	**1**	**2**	**3**	**4**
EDSS change, *N*′	51	35	25	17	513	392	286	235	1,962	1,564	1,225	1,025
Stable EDSS, %	82.4	71.4	64.0	64.7	79.5	74.7	69.6	70.2	79.1	73.8	70.1	68.1
EDSS improvement, %	13.7	20.7	24.6	23.4	14.0	17.9	19.2	19.1	11.9	13.6	15.1	17.2
EDSS progression, %	3.9	8.6	12.0	11.8	6.4	7.4	11.2	10.6	9.0	12.7	14.8	14.7
Clinical disease activity, *N*′	57	41	30	19	546	415	301	244	2,127	1,661	1,306	1065
Patients without activity, %	43.9	53.7	56.7	52.6	61.0	68.7	68.1	73.4	61.5	65.1	66.5	66.9

*EDSS, Expanded Disability Status Scale; N, number of patients in the total analysis population; N′, number of patients with available data*.

**Figure 3:**
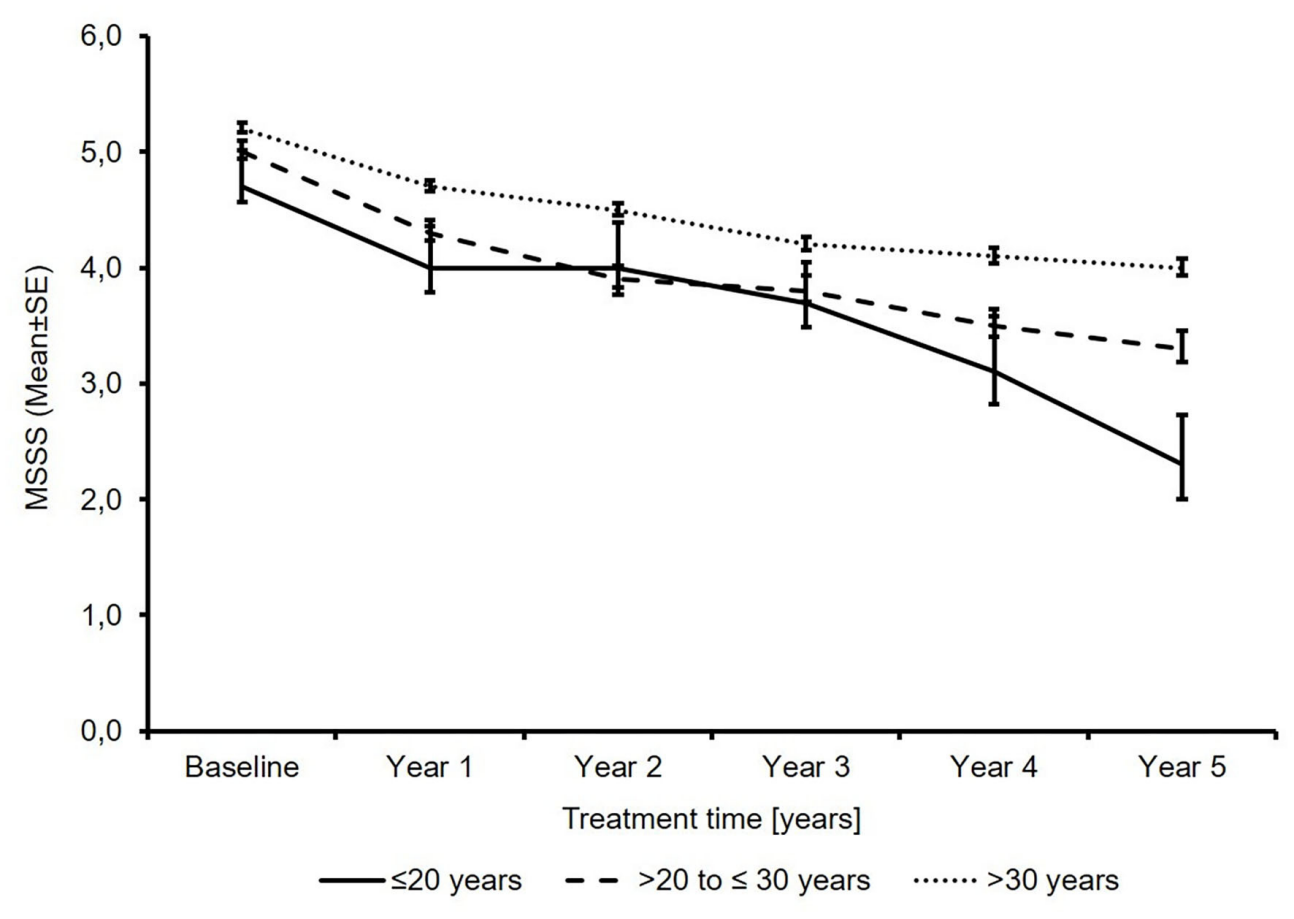
Total multiple sclerosis severity score (*N*′ ≤20 years: 71/51/34/26/17/12; *N*′ >20 to ≤30 years: 741/525/397/292/239/179; *N*′ >30 years: 2691/1953/1552/1216/1016/775; baseline/year 1/year 2/year 3/year 4/year 5).

The mean MSSS decreased in all age groups, with the lowest MSSS seen in the youngest patients (2.4 points and 1.7 points vs. 1.2 points; Figure 3).

**Figure 4:**
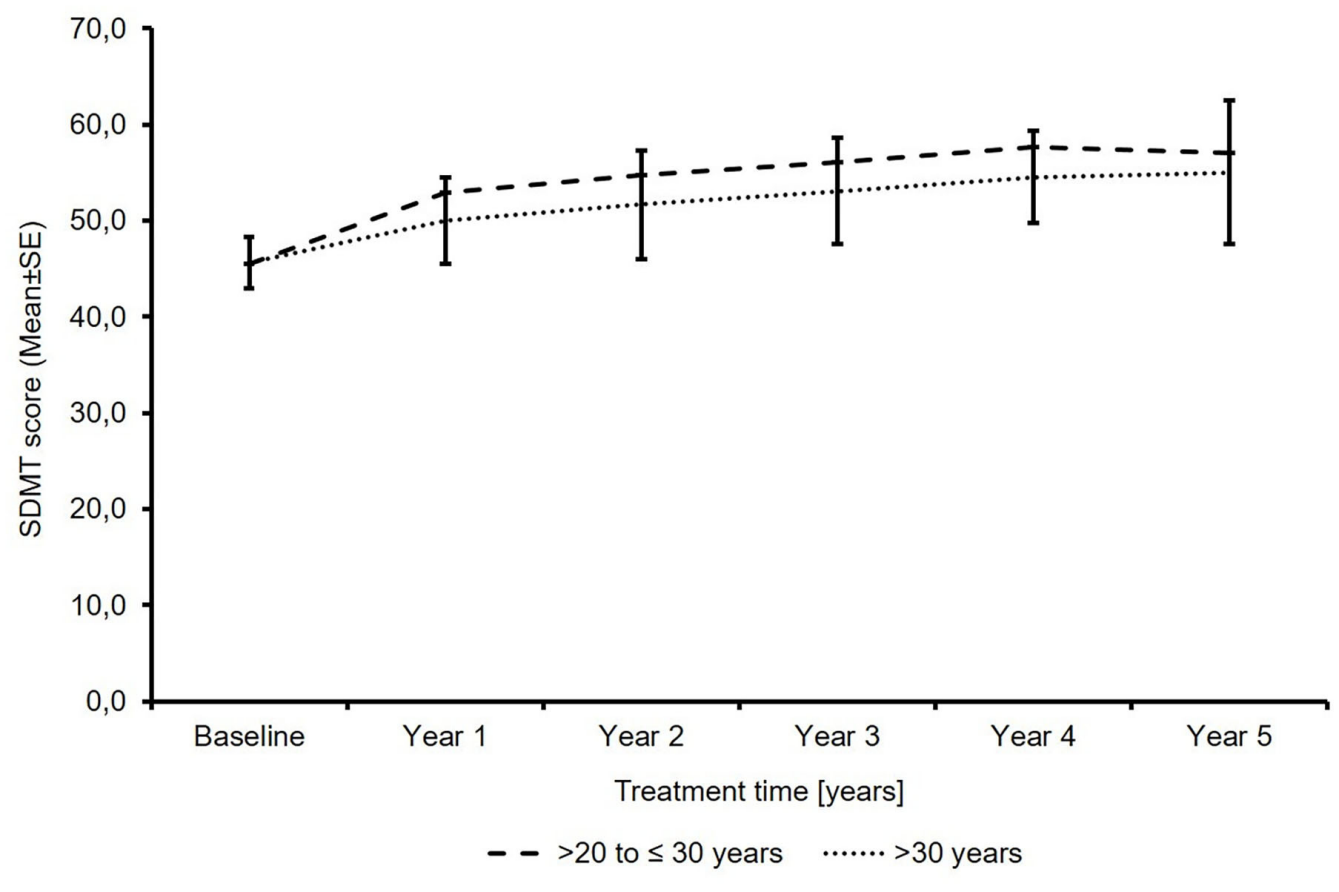
Total symbol digit modalities test score (*N*′ >20 to ≤30 years: 26/18/14/12/11/8; *N*′ >30 years: 189/173/151/120/89/67; baseline/year 1/year 2/year 3/year 4/year 5).

The SDMT total score increased from 45.5 points to 57.0 in patients ≤30 years of age and from 45.6 to 55.0 in patients >30 years of age. The mean change (± SD) in patients with available baseline and last visit data was 9.6 ± 12.8 (*n* = 7) and 8.1 ± 10.4 (*n* = 74) (Figure 4). No SDMT data were available in the subgroup of patients ≤20 years of age due to the small sample size of this subgroup, reflecting that SDMT is not a standard test in clinical practice.

Within 5 years of treatment, the effectiveness was deemed “good” or “very good” by ~80%, and “good” or “very good” tolerability was attested by over 90% of patients and physicians in all three subgroups (data not shown). The nature of reported adverse events is consistent with previous findings from clinical trials. The risk for infections does not differ between age groups with similar frequencies of lymphopenia (19.8 and 20.4 vs. 16.9%) and serious respiratory tract infections (1.2% and 2.5 vs. 2.86%) in all age groups.

## Discussion

The present descriptive analysis includes the final data of the PANGAEA study after the predefined maximum observation period of 5 years. It suggests that fingolimod provided long-term reduction of relapse rate, a stable or improved EDSS, and stable or improved cognitive function as assessed by SDMT in the majority of RRMS patients irrespective of age. The majority of patients in all age groups were free of any clinical disease activity, with the highest proportions reached in patients older than 20 years of age compared to younger patients. These results have to be interpreted in the context of age-dependent differences in patient characteristics, especially with respect to their baseline disease activity.

An analysis of the pivotal fingolimod trials by Gartner et al. has already assessed disease characteristics by age groups in patients from a clinical study setting (9). However, due to the inclusion and exclusion criteria in pivotal studies, the study populations do not cover the full range of patients treated in clinical practice. The present analysis of the PANGAEA study closes this gap and describes differences between age cohorts in a real-world setting. The same age cutoffs were used but with distinct groups for PANGAEA (≤20, >20 to ≤30 years, and >30 years), while Gartner *et al*. compared patients ≤20 and ≤30 years of age with the overall population. Consequently, patients in the oldest age group of PANGAEA were older on average and had a longer disease duration than the overall population of the pivotal studies. Despite these differences in the analyses, the PANGAEA results are very similar to what was found in the pivotal studies.

Over 90% of the young adults in the PANGAEA study showed relapse activity at baseline. In line with the present results, young adults were also found to have the highest clinical activity in terms of relapses at baseline in the pivotal fingolimod trials (9). This was expected, as natural history data indicate that younger patients have more frequent and more severe relapses than older patients and that relapse activity declines with increasing disease duration (4, 5). It can be assumed that, at this age, patients show a more inflammatory disease course.

Due to their higher disease activity, young patients have an urgent need for a highly efficacious treatment. The level of clinical disease activity despite treatment observed in PANGAEA suggests that insufficient disease control is more frequent in young patients than in older patients. This might, on the one hand, be due to the higher background disease activity and, on the other hand, due to the lack of authorized treatments for adolescent patients. Interestingly, the proportion of patients treated with beta-interferons as their last documented DMT was highest in the youngest age group. As only the last DMT has been documented in PANGAEA, it remains unclear whether the patients have received other DMT before. However, as until recently only beta-interferons were authorized for the treatment of RRMS in adolescents, it can be assumed that the lack of alternatives for this special population might at least have contributed and prevented adequate treatment optimization. Since its label was extended to the use in children and adolescents in 2019, fingolimod can be used for early intervention in young patients with highly active RRMS. The previous analyses of study data of the pivotal trials have shown that fingolimod significantly reduced the ARR and the number of new T2 lesions compared to placebo and interferon-beta 1a in young adult patients (9) as well as in children and adolescents (8). According to the reduction of the ARR and lesion load in the clinical study setting, fingolimod adequately addresses these pathological processes in patients with early-onset MS. The present data of the PANGAEA study suggest that the effective relapse prevention observed in young adults in the clinical study setting translates into clinical praxis. As the proportion of relapse-free patients increased by ~60 percentage points in each group, it can be assumed that RRMS patients benefit from fingolimod treatment to the same extent with respect to the reduction of relapse activity irrespective of their age. The higher underlying relapse activity in the younger group might be the reason for the lower overall proportion of relapse-free patients compared to the older groups.

Although younger patients have more frequent and more severe relapses, they often completely recover from their relapses. The present PANGAEA results on young adults indicate that a higher proportion of patients is able to reach disability improvement as measured by EDSS and that the positive treatment effect on cognitive function is more pronounced compared to older patients. This might be due to a higher compensatory capacity at this young age, which then continuously declines with increasing age (14). In line with this, EDSS progression probability is higher in older patients, and although the relapse rate was not higher in these patients, relapse-related EDSS worsening was more frequent (15). It has to be pointed out that the higher EDSS progression probability might be confounded by a higher disease duration in older patients and is not solely age-driven. Nevertheless, it can be assumed that an increasingly incomplete recovery from relapses with older age and higher disease duration due to a decrease in compensatory activity hampered the effectiveness of fingolimod in older patients. The specific processes of such impairment in older MS patients are not fully understood yet, but cell senescence with oxidative stress, decreased intrinsic autophagy, and reduced neurotrophic support might play a role (16). Therefore, immunosenescence could have affected the effectiveness in older subjects.

The recovering capacities at a young age still do not allow for a delay in treatment initiation (17). Roving EDSS analysis from PANGAEA data showed that EDSS progression unrelated to relapses occurred to a similar extent in all age groups, supporting the concept that chronic disease progression is present already from disease onset and significantly contributes to overall disability progression (18). Therefore, undelayed treatment initiation and optimization are highly important in patients of any age, and the PANGAEA study data support the use of fingolimod in all age groups, including young adults. It is essential especially for young patients not only to prevent disability progression in terms of motor function but also to assure stable cognitive functionality. This phase of life is very demanding as, for example, academic studies, vocational education and training, and career entry and progression require full cognitive capacities. The SDMT is a strong predictor of vocational status (19), and an SDMT worsening of three points is clinically meaningful and results in reduced working capabilities and responsibilities (20). Hence, a slight deterioration can already have a marked impact. Early treatment intervention can help to prevent slight but meaningful deterioration at an early stage of the disease, and long-term treatment outcomes potentially benefit from the synergism of an effective disease activity control and a high compensatory activity. A higher effectiveness in terms of disability improvement in younger patients of the PANGAEA study might therefore reflect the benefits from early treatment initiation at a younger age and earlier diagnosis.

In line with this, recent analyses from pivotal fingolimod trials indicate that immediate treatment is superior to delayed treatment in young adults in terms of long-term benefits in disease activity and disability progression (21). High-efficacy treatment initiation within 2 years of MS onset compared to a start within 4–6 years after disease onset was associated with less disability (22). A propensity score-matched comparative analysis of PANGAEA and a non-interventional study on the use of beta-interferon or glatiramer acetate found that switching to fingolimod early is more effective in patients with active disease than continuing beta-interferon or glatiramer acetate (23). Further analyses of real-world data, including the PANGAEA study data, provide good evidence of its effectiveness in the treatment of active MS (24). The results of a recent multicenter cohort study even support the preference of newer disease-modifying drugs, including dimethyl fumarate, fingolimod, teriflunomide, natalizumab, rituximab, ocrelizumab, and alemtuzumab, over beta-interferon or glatiramer acetate for the initial treatment of pediatric patients and clinically isolated syndrome (25). Furthermore, the risk of conversion to a secondary progressive disease course was found to be significantly reduced under initial treatment with fingolimod, natalizumab, or alemtuzumab (26). These findings may contribute to the current change in mindset toward an early intervention with efficacious drugs. An analysis of baseline characteristics in the PANGAEA study in comparison to the characteristics in a similar successor study, PANGAEA 2.0, indicate that patients were switched to fingolimod at an earlier stage of their disease (27).

Apart from the differences in their baseline disease activity, the age groups in the PANGAEA study also differed with respect to comorbidities, which, in general, were more frequent in older patients. The prevalence of psychiatric disorders, vascular disorders, nervous system disorders, endocrine disorders, as well as musculoskeletal and connective tissue disorders was at least twice as high in patients older than 30 years compared to young adults <20 years of age. The pattern of comorbidities observed in PANGAEA is in line with what has been previously reported for MS patients and what has been shown to be significantly associated with an increase in treatment switches due to intolerance and with a stronger EDSS increase (28). An increased cardiovascular risk, as estimated by the Framingham score, was significantly associated with a higher risk for relapse, for reaching EDSS 6.0 and for treatment escalation (29). Furthermore, psychiatric disorders are known to have a strong impact on the quality of life, fatigue, physical disability, and cognitive performance as well as medication adherence (30). In PANGAEA, older patients had more comorbidities and a higher risk for EDSS progression. With respect to the possible influence of comorbidities on MS symptoms and treatment outcomes, the impact of age-dependent comorbidity prevalence on the present comparison has to be considered. To what extent the comorbidities in older PANGAEA patients affected previous treatment switches and present treatment outcomes cannot be estimated.

From a safety point of view, fingolimod can be initiated immediately also in early-onset MS patients. According to the present PANGAEA analyses, physicians as well as patients rated the effectiveness and tolerability to be good or very good. This is in line with the results in young adults from the pivotal studies, which reported a safety profile similar to that of placebo and consistent with that observed in the overall adult population (9). It has been previously shown that patient-perceived good effectiveness and tolerability also translates into very low frequencies of treatment interruptions or discontinuations (31). In the PANGAEA study, about half of the patients discontinued study documentation prematurely, but only approximately one-third of these discontinuations were associated with a lack of effectiveness, disease progression, or lack of tolerability. Drop-outs due to a lack of effectiveness were more frequent in the youngest group compared to older patients. On the other hand, fewer patients <20 years reported disease progression or relapse as a reason for discontinuation. Taken together, the rate of patients who discontinued due to either a lack of effectiveness, disease progression, or relapse is similar. However, as multiple answers were possible, there might be an overlap of patients between both dropout categories.

Overall, this analysis of the PANGAEA study suggests disease- and non-disease-specific differences between younger and older patients, with higher disease activity in younger patients and higher levels of physical disability and more comorbidities in older patients. Despite these differences, fingolimod reduced the overall clinical disease activity as well as the relapse rate, slowed disability progression, and was well-tolerated irrespective of age. The present real-world data suggest that fingolimod can be used for treatment optimization in young patients already at an early stage of the disease.

## Data Availability Statement

The datasets generated and/or analyzed during the current study are available from the corresponding author on reasonable request.

## Ethics Statement

Ethical review and approval was not required for the study on human participants in accordance with the local legislation and institutional requirements. The patients/participants provided their written informed consent to participate in this study.

## Author Contributions

TZ, HA, JH, LK, ML, CL, and SS contributed to data collection. TZ contributed to the study conception and design. Analyses were planned by TZ, US-T, and BE. The first draft of the manuscript was written by TZ and US-T with the assistance of a medical writer, and all authors commented on the previous versions of the manuscript. All authors contributed to the article and approved the submitted version.

## Conflict of Interest

TZ has received personal compensation for participating on advisory boards, trial steering committees, and data and safety monitoring committees as well as for scientific talks and project support from Bayer HealthCare, Biogen, Celgene, Genzyme, Merck, Novartis, Roche, Sanofi, and Teva. HA has received travel grants, speaker's honoraria, and consultancy fees from Teva, Merck Serono, Genzyme, Sanofi, Novartis, Bayer, and Biogen. JH has received honorarium from Biogen Idec, Merck Serono, Bayer Schering, Teva-Aventis, Novartis, and Octapharma. LK received compensation for serving on scientific advisory boards for Genzyme and Novartis. She received speaker honoraria and travel support from Novartis, Merck Serono, and CSL Behring and receives research support from Novartis and Biogen. ML has received research support from Novartis. CL has received travel grants, speaker's honoraria, financial research support, and consultancy fees from Teva, Merck Serono, Genzyme, Sanofi, Novartis, Bayer, and Biogen. SS has received speaking honoraria and travel compensations and has served on advisory boards for BayerVital, Biogen, MerckSerono, Novartis, and Teva. BE and US-T are employees of Novartis Pharma GmbH, Nuremberg, Germany. The authors declare that this non-interventional study was sponsored and funded by Novartis Pharma GmbH. The role of the sponsor and funder included protocol development, study administration, data management, data analysis, and manuscript preparation. Novartis Pharma GmbH further funded the medical writing support.
